# Effectiveness of Self-Care Education for Chronic Neck Pain: A Systematic Review and Meta-Analysis

**DOI:** 10.3390/healthcare11243161

**Published:** 2023-12-13

**Authors:** Geraldine Valenza-Peña, Javier Martín-Núñez, Alejandro Heredia-Ciuró, Alba Navas-Otero, Laura López-López, Marie Carmen Valenza, Irene Cabrera-Martos

**Affiliations:** Department of Physiotherapy, Faculty of Health Sciences, University of Granada, Av. De la Ilustración, 60, 18016 Granada, Spain; valenzagera@hotmail.com (G.V.-P.); javimn@ugr.es (J.M.-N.); ahc@ugr.es (A.H.-C.); cvalenza@ugr.es (M.C.V.); irenecm@ugr.es (I.C.-M.)

**Keywords:** chronic neck pain, self-care education program, systematic review, meta-analysis

## Abstract

Self-care programs for chronic neck pain are relevant to everyday life and can lead to long- term improvement. More studies on their effectiveness, key components and appropriate duration are needed. The aim of this study was to determine the effectiveness of self-care programs for patients with chronic neck pain. A systematic review and meta-analysis of randomized controlled trials was conducted according to the PRISMA guidelines. After searching in PubMed, Web of Science, Scopus and ScienceDirect, eleven studies met the inclusion criteria. Self-care education interventions typically consisted of education (i.e., pain neuro-science education or general educational concepts) accompanied by exercise or manual therapy. The most frequent components were addressing physical and psychological symptoms and engaging in self-care strategies. The least frequent ones were monitoring and recording symptoms and discussing with providers of medical care. The duration of the interventions ranged from three sessions to six months. Finally, individual and supervised modalities were the most frequent. After pooling the data, a meta-analysis was carried out according to four variables (i.e., pain, disability, kinesiophobia and catastrophization) and showed significant results (*p* < 0.05) in favor of self-care interventions. This systematic review and meta-analysis suggests that self-education interventions improve pain, psychological pain-related variables and disability in patients with chronic neck pain. The most frequently used components were addressing physical and psychological symptoms and engaging in self-care strategies. Future trials should focus on including other components, such as discussing symptoms with providers of medical care or self-monitoring symptoms. Additional areas of focus include more homogeneous doses and comparator treatments, as well as studies with better evidence to reach more solid conclusions.

## 1. Introduction

Neck pain represents a substantial health and economic burden and is one of the leading causes of years lived with disability worldwide [1]. The prevalence and incidence of this condition are variable and range from 5.2% to 8.56% in Spanish population-based surveys [2,3]. Moreover, the burden of this condition is accompanied by the fact that, in many cases, patients may develop persistent or recurrent pain [4,5]. In fact, the transition from acute or subacute neck pain to chronic neck pain has been associated with non-modifiable factors—including age, gender and comorbidity with other disorders—and modifiable factors—such as psychosocial aspects, sleep troubles, job stress and work-related posture [6]. Chronic neck pain can seriously affect the quality of life, increasing the presence of disability [7], motor impairment [8] and fear-avoiding behaviors [9]. These aspects severely impact psychological, behavioral, mental and psychosocial components of health. Maladaptive pain coping behaviors, such as fear avoidance and pain catastrophizing, have been described as determining aspects of chronic pain in the same line as other comorbidities, such as depression and sleep deprivation [10]. 

The most recent systematic reviews (published in 2022 and 2023) concluded that a range of interventions for chronic neck pain, such as electrotherapy [11,12], massage [13], psychological intervention [14], pain education [15], neck manipulation and mobilization [16] or exercise [17,18], can have a variable degree of effectiveness. Those reviews provide small to moderate evidence due to the difficulty of including different patient etiologies and profiles. 

In their systematic review, Sterling et al. (2019) [1] concluded that conventional rehabilitation approaches have limited efficacy, and a significant paradigm shift is necessary to improve this situation. In the same vein, other authors have drawn similar conclusions on conservative treatments and there is high heterogeneity between current guideline recommendations. This leads to uncertainty about which treatment options are likely to be the most effective [19,20]. 

Specifically, because of difficulties reaching a consensus, numerous large population studies [21,22] have been conducted with multimodal programs, showing different degrees of clinical usefulness. Multimodal programs have paid some attention to the potential role of behavioral techniques in the treatment of chronic musculoskeletal pain [23]. The inclusion of non-pharmacological interventions aimed at improving the physiological and psychological state of patients by providing them with education programs can improve the wellbeing of individuals with musculoskeletal pain [24]. An example of this is self-care education based on self-care management practices, including opting for a healthy lifestyle, self-monitoring, assessing symptoms, evaluating symptom severity and determining treatment alternatives [25]. Additionally, self-care management practices have proven to be easily included in daily clinical practice in combination with other conservative treatments.

Chronic pain, like all chronic conditions, requires day-to-day management by patients. Self-care education interventions can target behavioral factors, day-to-day management and self-efficacy-enhancing strategies, so they are adequate for chronic pain conditions [26]. Specifically, some studies have reported that self-care education programs can efficiently reduce the occurrence and severity of symptoms and improve quality of life [27]. 

However, there is no systematic review and meta-analysis on self-care education programs for patients with chronic neck pain, despite a clear need for a summary of existing evidence. Self-care education programs are relevant to everyday life and can lead to long-term improvement, but information on their effectiveness, components and duration is limited. Thus, this systematic review with a meta-analysis was aimed at synthesizing the existing evidence about self-care education programs in patients with chronic neck pain.

## 2. Methods

The systematic review adhered to the principles outlined in the Preferred Reporting Items for Systematic Reviews and Meta-Analyses (PRISMA) statement [28] and followed the Cochrane Collaboration guidelines for reviewing interventions [29]. The protocol for this systematic review was registered in PROSPERO in 2022 (registration number: CRD42022303416).

A comprehensive literature search for articles indexed in PubMed, Web of Science and Scopus encompassing randomized controlled trial databases from their inception to July 2023 was conducted. The search strategy in MEDLINE involved the following steps: (1) developing keywords by analyzing the relevant terms used in existing systematic reviews; (2) a comprehensive exploration of the MeSH Database pertaining to terms such as “neck pain”, “self-care program”, “chronic neck pain” and “self-management education program”; and (3) obtaining expert guidance and undergoing specialist review. This search strategy was rigorously tested and refined to ensure its effectiveness for this review. Subsequently, this strategy was adapted for use in other databases (Table 1).

The references of relevant reviews were examined to identify additional studies that might be eligible for inclusion in this review. Articles written in Spanish or English were included.

The PICOS (Population, Intervention, Comparison, Outcomes and Study design) model was used [30] to formulate the research question. The inclusion criteria were as follows: (1) individuals with chronic neck pain; (2) self-care education programs isolated or added to standard care; (3) comparison with a control intervention that did not include education; (4) assessment of pain, disability and psychosocial pain variables as outcomes; and (5) inclusion of only randomized clinical trials.

The exclusion criteria were (1) designs other than randomized clinical trials; (2) patients with non-chronic neck pain included; (3) interventions that did not include education; and (4) articles written in languages other than Spanish or English. 

The classification of a self-care education intervention was made in accordance with the Chronic Care Model and the definition put forth by Yahaya et al. [25]. The self-care education intervention was developed using the intervention-mapping approach [31]. It was designed to alleviate psychological distress, increase patient activation and evaluate treatment-related concerns. The intervention program developed illustrated the relationship between actions and patient outcomes using suggested elements such as patient problems and intervention measures. According to Yahaya et al., a self-care education intervention is tailored to the needs of the individual and focuses on four self-care goals, addressing physical and psychological symptoms, engaging in self-care strategies, quickly monitoring and recording symptoms, and reporting and discussing symptoms with providers of medical care. Articles that met a minimum of two of the four objectives described previously were included.

After all studies were retrieved from the databases, duplicates were removed using Mendeley. 

Two independent authors (G.V. and C.V.) performed a first screening of the title and abstract. In the second screening, articles were selected according to the full text. Once the articles were selected, data extraction and quality assessment were carried out. A third reviewer (J.M.) was responsible for resolving any disagreement between the two main reviewers.

We applied the Cochrane risk-of-bias tool for cluster-randomized trials (RoB 2 CRT) [29], which comprises five domains and an overall judgment. These domains are as follows: (1) bias arising from the randomization process; (2) bias resulting from deviations from the intended interventions; (3) bias due to missing outcome data; (4) bias in the measurement of the outcome; and (5) bias in the selection of the reported result [32]. Based on the responses (yes; probably yes; probably no; no; not applicable; no information) to a series of signaling questions, the judgment options within each domain include “low risk of bias”, “some concerns” or “high risk of bias”. If a study was deemed to have a low risk of bias across all domains for a given outcome, we considered it to have an overall low risk of bias for that outcome. If a trial was assessed as having a high risk of bias in one domain or “some concerns” in multiple domains (three or more) for a given outcome, we concluded that it had an overall high risk of bias for that outcome. If a study was found to raise some concerns in at least one domain for a given outcome but did not have a high risk of bias in any one domain, we categorized it as having some concerns.

The evaluation reviewed findings and grouped them into categories based on the Grading system for Rating Guidelines, Testing and Appraisal of Evidence and Results (GRADE). This structure considers five aspects: research design, imprecision, indirectness, inconsistency and publishing bias [33].

The available information was divided into four clear categories, as follows: (a) very low quality, indicating a circumstance where any analysis of impacts is highly doubtful, with three of the five areas failing to meet the standards; (b) low quality, where further investigation is highly likely to significantly affect our confidence in the estimate of impacts and could potentially lead to changes as additional investigation could alter our understanding; (c) moderate quality, meaning that further research is likely to significantly affect our certainty in the impact assessment and may result in changes because one of the five areas does not meet the principles; and (d) high quality, where additional research is extremely unlikely to affect our certainty in the impact assessment and all five areas absolutely meet the principles [34].

The assessment of the five domains adhered to the standards set by GRADE. For the study style domain, proposals were downgraded by one level where there was ambiguity or a high risk of bias, combined with major limitations in the impact assessment. With regard to unpredictability, the proposals were additionally lowered by one level if the quantitative assessments showed a large fluctuation between the studies, if the safety intervals showed little overlap or if I^2^ indicated a large or very large heterogeneity. In the domain of indirectness, the recommendations were downgraded if there were considerable differences in the interventions, the study populations or the results. In the area of imprecision, recommendations were downgraded by one level if there were fewer than 400 participants for continuous data [35].

When possible, study results were pooled, and a meta-analysis was undertaken using Review Manager software (RevMan version 5.1, updated March 2011). Due to the clinical heterogeneity of the studies included, this meta-analysis was limited. The I^2^ statistic was used to determine the degree of heterogeneity, where the percentages quantified the magnitude of heterogeneity: 25% = low, 50% = medium and 75% = high heterogeneity. Using this scale, if I^2^ was 50%, a random effects model was used. All the outcomes included were continuous data outcomes (i.e., pain intensity, disability, kinesiophobia and catastrophizing); the mean difference with a 95% confidence interval (CI) was used in the analysis. Forest plots were generated to illustrate the overall effect of interventions.

## 3. Results

**Figure 1:**
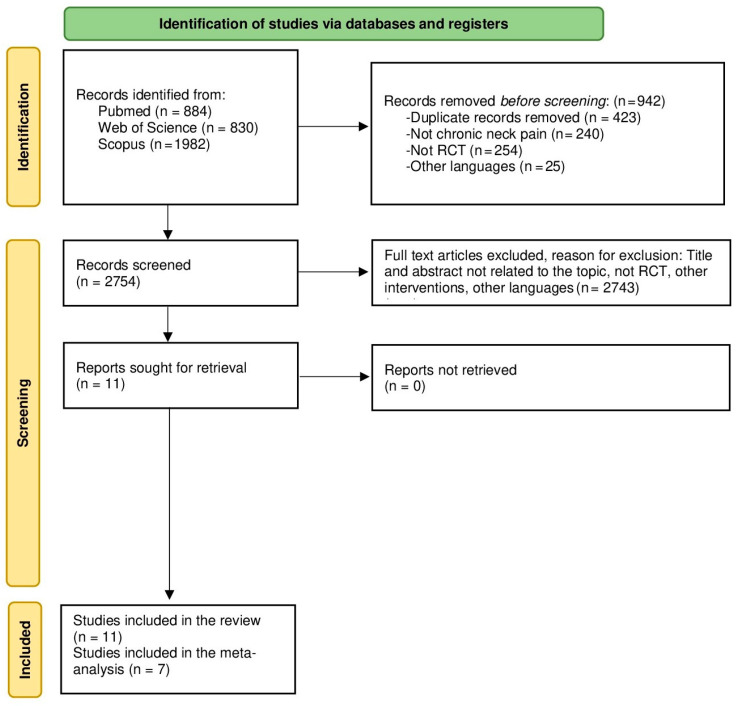
PRISMA flowchart of the literature search and study selection.

The search outcomes and the final selection of studies are presented in Figure 1. At the outset, a total of 3700 search results were collected from the databases and secondary searches were conducted within the specified search dates outlined in the methods. Following the assessment of the titles and abstracts, articles that did not meet the inclusion criteria were excluded. A comprehensive review of the full texts resulted in 15 articles being quantified; in total, 21 studies remained after eliminating those that did not meet the inclusion criteria [36,37,38,39,40,41,42,43,44,45,46]. The reasons for the exclusion of the articles are specified in Figure 1.

A total of 854 participants with chronic neck pain receiving self-care education interventions was included. Study design, sociodemographic and clinical characteristics of patients and quality scores are detailed in Table 2.

Most participants included in the studies were female and were middle-aged (30 to 50 years old). Pain intensity was reported in only four studies and typically ranged from 5 to 7 on a Visual Analogue Scale (VAS). Most patients reported a duration of pain from 1.64 ± 0.65 to 7.98 ± 6.46 years.

Table 3 shows the intervention characteristics and main conclusions obtained for each study.

Among the studies included, four [38,40,44,45] compared self-care education in isolation with other interventions; the remaining articles [38,40,44,45] compared self-care education combined with physical therapy (i.e., therapeutic exercise or manual therapy) to other interventions. 

Self-care education interventions typically consisted of education (i.e., pain neuroscience education or general educational concepts). Additionally, five studies [36,37,41,42,46] compared three intervention groups and the other six included two intervention groups [38,39,40,41,44,45].

The duration of the interventions ranged from 4 to 12 weeks in most studies, but lasted for 1 week in two studies [38,39]. Most interventions were supervised (individually or in groups) and only three studies [41,43,44] included telehealth interventions (i.e., phone calls or information sent via email).

Pain was measured with the VAS, the Brief Pain Inventory (BPI) or the Numeric Pain Rating Scale (NPRS). The most frequent tool used to assess disability was the Neck Disability Index (NDI). Psychosocial variables included the Tampa Scale of Kinesiophobia (TSK), the Fear Avoidance Beliefs Questionnaire (FABQ) and the Pain Catastrophizing Scale (PCS).

Results showed significant improvements in favor of self-care education interventions isolated or in combination with physical therapy. When compared, self-care education interventions added to physical therapy showed better results than self-care education or physical therapy interventions alone.

**Figure 2:**
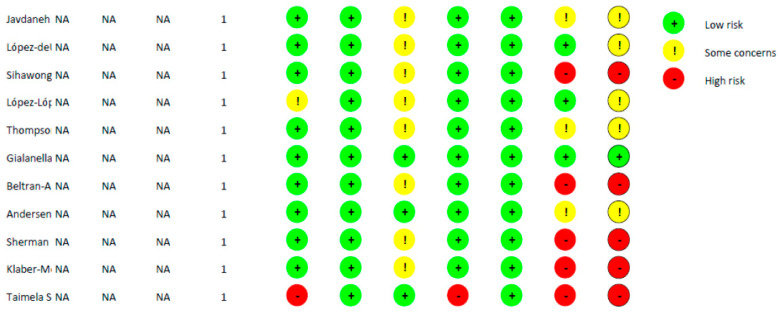
Quality assessment of the studies included.

Cochrane Risk of Bias Assessment results are shown in Figure 2.

The quality assessment using the Cochrane Risk of Bias Assessment showed high risk or some concerns for all the studies. 

Table 4 shows the self-care components in each study included.

As seen in Table 4, all the studies selected addressed physical and psychological symptoms, including information about the emotional response to pain, anxiety, frustration and fear of damage, as well as modifying erroneous beliefs about pain and disability, providing coping strategies and improving patient self-efficacy through a graded activity.

In addition, all the studies included self-care strategies such as lifestyle advice and support or information about the condition and/or its management. Three [38,41,46] of the eleven studies included symptom monitoring and only two studies [39,41] included reporting and discussing symptoms through regular visits to healthcare professionals.

According to the GRADE recommendations, there was low-quality evidence regarding the effects of self-care education for chronic neck pain on pain, disability, kinesiophobia or catastrophization; although all of the studies were RCTs, these studies were downgraded due to the risk of bias (performance and detection bias), inconsistency (range I_2_ = 72 to 97%) and imprecision because of a sample size of less than 400. Low-quality evidence was found regarding the isolated effects of self-care strategies on pain and disability due to the risk of bias (performance and detection bias), inconsistency (range I_2_ = 72 and 97%) and imprecision (n = 293). Likewise, there was low-quality evidence regarding the effects of self-care education combined with physical therapy on pain, disability, kinesiophobia and catastrophization, being downgraded due to inconsistency (I_2_ = 72–93%) and imprecision (n = 170).

### Results Obtained in the Meta-Analysis

**Figure 3:**
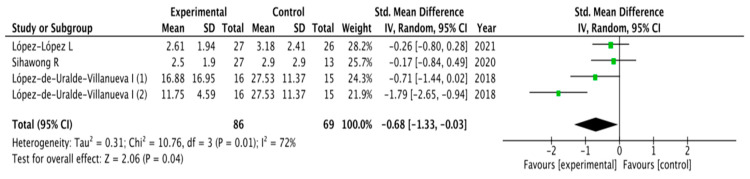
Forest plot for pain.

**Figure 4:**
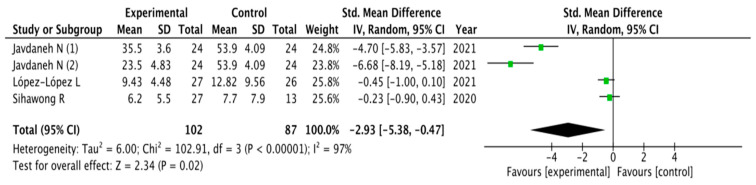
Forest plot for disability.

**Figure 5:**
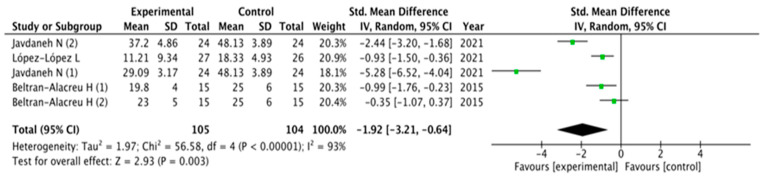
Forest plot for kinesiophobia.

**Figure 6:**
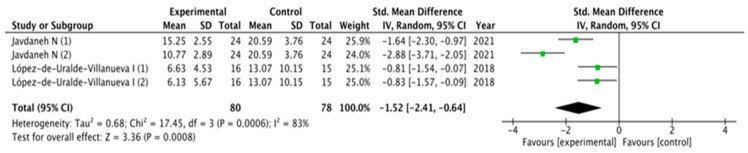
Forest Plot for catastrophization.

The results obtained in relation to pain, disability, kinesiophobia and catastrophization were analyzed across the different studies included. Figure 3, Figure 4, Figure 5 and Figure 6 show the different analyses.

For pain, the pooled mean difference (MD) showed a significant overall effect in favor of SCE interventions when compared to physical therapy or exercise (MD = −0.68; 95% CI = −1.33, −0.03; *p*= 0.04). Heterogeneity was moderate (I^2^ = 72%).

Disability showed similar results in favor of SCE interventions: (MD = −2.93; 95% CI = −5.38, −0.47; *p*= 0.02). Heterogeneity was high (I^2^ = 97%). Kinesiophobia and catastrophization were analyzed and showed significant overall effects in favor of SCE interventions (MD = −1.52; 95% CI = −2.41, −0.64; *p*= 0.0008 and MD = −1.92; 95% CI = −3.21, −0.64; *p* = 0.003, respectively). Heterogeneity was high (I^2^ = 83%) for catastrophization and moderate for kinesiophobia (I^2^ = 93%).

## 4. Discussion

Our findings provide support for the effectiveness of self-care education interventions in the treatment of patients with chronic neck pain. However, it is important to interpret the results of the literature with caution, given the variations in the components included in self-care education programs, the additional techniques included in the interventions (e.g., exercise and physical therapy) and variations in the implementation of self-care education programs. 

Most participants included in this systematic review and meta-analysis were females aged between 30 and 50 years. According to the scientific literature, approximately 50% of chronic pain conditions, including chronic neck pain, have a higher prevalence in women [47]. Sex and gender are critical factors that condition the pain experience and therapeutic strategies [48]. It is widely acknowledged that men and women exhibit different physiological and behavioral reactions to pain. Gender dimensions play a significant role across various health domains, influencing the socially contextualized encounter and manifestation of physical symptoms, attitudes and beliefs regarding health, participation in health-related behaviors and interactions within the healthcare system [49,50]. These psychosocial factors associated with chronic neck pain should be considered in the treatment of these patients. It is essential to integrate and assess strategies grounded in a biopsychosocial approach. These strategies should encompass cognitive and behavioral elements related to the perpetuation of neck pain. This approach aims to promote the development of active pain-coping skills and self-pain management, thereby diminishing pain-induced disability [51].

Among the self-care education interventions included in this systematic review, behavioral and pain neuroscience education was the most commonly utilized, with physical therapy and exercise frequently serving as control treatments. In addition, some of the articles included combined self-care education programs with other interventions, such as exercise, showing favorable results in pain and psychological pain-related variables. 

Other studies found larger effect sizes in the reduction of fear of movement when pain neuroscience education and physical exercise were combined [52,53]. In their systematic review and meta-analysis, Rathnayake et al. (2021) [54] concluded that including exercise in self-care interventions showed a moderate yet notable positive impact, spanning short-, intermediate- and long-term periods, on reducing both pain and disability in patients with chronic lower back pain.

The duration of interventions ranged from 4 to 12 weeks in most studies. These results are in line with similar interventions in other pathologies. In the systematic review and meta-analysis conducted by Du et al. 2020 [55], the authors concluded that self-management programs with durations ≤ 8 weeks demonstrated a better immediate effect on pain in patients with chronic lower back pain.

The most frequently used components in the studies included were addressing physical and psychological symptoms and engaging in self-care strategies. These components included details regarding the emotional reaction to pain encompassing anxiety, frustration and fear of exacerbation, alongside rectifying misconceptions about pain and disability, offering coping mechanisms and enhancing patient self-confidence through a gradual engagement in exercise activities. Furthermore, all the articles included self-care tactics, such as lifestyle guidance, support or education regarding the condition and/or its effective management. Nevertheless, most studies missed relevant components such as discussing symptoms with providers of medical care or self-monitoring symptoms.

Self-monitoring is a crucial element in patients’ self-care [56]. It presents an opportunity to raise awareness regarding symptoms, bodily sensations, daily routines and cognitive patterns, providing valuable information for subsequent actions [57]. The potential benefits of self-monitoring seem encouraging: the existing literature suggests it can enhance self-care practices, symptom control and disease management, potentially resulting in decreased complications, improved coping mechanisms and attitudes towards the illness, establishment of practical goals and an overall improved quality of life [58].

The authors of future studies should consider including these components in interventions with these patients.

The results of this systematic review and meta-analysis are consistent with previous systematic reviews conducted in the population with chronic pain, as demonstrated in several studies [59,60,61]. Our paper holds significance and fills a current need as it provides updated information and delivers a high-quality level of evidence. The outcomes of our systematic review indicate that self-care education programs can yield positive effects on pain, disability, kinesiophobia and catastrophizing.

When analyzing the results of the comparison between self-care education and other interventions, other reviews on comparable interventions such as self-management obtained similar results in other pathologies.

Lorig and Holman [62] defined the tasks involved in the self-management of a chronic condition to enhance the quality of life. These tasks include managing medical interventions, such as using medication appropriately; using cognitive and behavioral strategies to manage symptoms; role modification; and strategies to deal with the emotional consequences of a chronic condition. Interestingly, our analysis showed results in favor of self-care education when considering studies that only included educational components. 

The daily challenges in individuals with chronic neck pain can be different for each individual and may change over time. Thus, transferable skills such as problem solving, decision making, resource identification and communication skills are invaluable. In this regard, the study interventions only included some of the skills (e.g., therapeutic exercise, pain education) necessary for patients with chronic neck pain to deal with their symptoms in the long term.

When analyzing the results obtained in this meta-analysis regarding pain variables, the mean standard difference (MSD) was 0.68 for our included studies [63]. Similar meta-analyses in back pain and conservative treatment have shown results in the same line when comparing interventions with other treatments. With respect to kinesiophobia, this meta-analysis showed a MSD of −1,92, which is higher in comparison to similar meta-analyses on chronic pain [64].

The catastrophization evaluated by the Pain Catastrophizing Scale showed a MSD of −1.52 in this meta-analysis. This result was not clinically relevant since the change was less than 6.48, which is the minimal clinical importance difference on the PCS scale [65]. In addition, the MSD obtained in this meta-analysis for neck disability is similar, although slightly lower in magnitude compared to another meta-analysis [66]. 

Furthermore, self-care education requires the ability to assess one’s circumstances and available resources, and to make informed decisions [67]. The typically supervised and individually tailored studies included in this context align with the recommendations presented by Bandura et al.: emphasizing the significance of practicing and mastering a task or skill, observing peers as they model the skill, receiving feedback and support and striving to enhance one’s emotional wellbeing [67].

Our review has some limitations; first, many intervention groups only used education as a self-care education intervention combined with physical therapy and provided little description of the dosage, intensity and individualization of the treatment. 

Second, the profile of the subjects was limited to pain duration and severity prior to the studies. However, some information about the degree of disability before interventions or the specific etiology of the chronic neck pain could be relevant. Third, the heterogeneity of the studies makes it difficult to draw solid conclusions, and further studies are needed to delve deeper into this topic. Another limitation is the absence of a funnel plot or statistical test in the evaluation of publication bias. Nevertheless, the Cochrane Risk of Bias Assessment is a reliable and validated tool for the assessment of the risk of bias. The lack of consistent reporting of pain intensity across the studies is another limitation. A more consistent and comprehensive reporting of pain intensity across all studies is something the authors of future articles should strive for. 

## 5. Conclusions

This systematic review and meta-analysis suggests that self-education interventions improve pain, psychological pain-related variables and disability in patients with chronic neck pain. The results were significant for pain severity, disability, kinesiophobia and catastrophization. The most frequently used components comprised addressing physical and psychological symptoms and engaging in self-care strategies. Future trials should include other components, such as discussing symptoms with providers of medical care or self-monitoring symptoms. Additional areas of focus could include more homogeneous doses and comparator treatments, as well as studies with better evidence, to reach more concrete conclusions.

## Figures and Tables

**Table 1:** Search equation in each database and results.

Database	Search Equation	Results
PubMed	((self-care[MeSH Terms]) AND (patient participation[MeSH Terms]) AND (health promotion[MeSH Terms]) AND (patient autonomy[MeSH Terms]) AND “self care” OR “self-care” OR “self manage” OR “self-manage” OR “self efficacy” OR “self-efficacy” OR “home base” OR “home-base” OR “health education” OR “patient education” OR “patient participation” OR “health communication” OR “health promotion” OR “self concept” OR “self-control” OR (MH “self concept”) OR “self-regulation” OR “patient autonomy*” OR (MH “patient compliance”) OR (MH “health behavior”) OR “health attitude” OR “illness attitude” OR “patient attitude*” OR (MH “choice behavior”) OR (MH “illness behavior“) OR “self management” “disease management” AND “chronic pain” OR “musculoskeletal pain” OR “myalgia” OR “neck pain” OR “musculoskeletal pains” OR “muscle pain” OR “neck pains” OR “cervical pain” OR “cervical pains” OR “myofascial pain syndrome” OR “trigger point”)	884
Web of Science	TS = (“self care” OR “self-care” OR “self manage” OR “self-manage” OR “self efficacy” OR “self-efficacy” OR “home base” OR “home-base” OR “health education” OR “patient education” OR “patient participation” OR “health communication” OR “health promotion” OR “self concept” OR “self-control” OR (MH “self concept”) OR “self-regulation” OR “patient autonomy*” OR (MH “patient compliance”) OR (MH “health behavior”) OR “health attitude” OR “illness attitude” OR “patient attitude*” OR (MH “choice behavior”) OR (MH “illness behavior“) OR “self management” “disease management” AND “chronic pain” OR “musculoskeletal pain” OR “myalgia” OR “neck pain” OR “musculoskeletal pains” OR “muscle pain” OR “neck pains” OR “cervical pain” OR “cervical pains” OR “myofascial pain syndrome” OR “trigger point”)	830
Scopus	TITLE-ABS-KEY (“self care” OR “self-care” OR “self manage” OR “self-manage” OR “self efficacy” OR “self-efficacy” OR “home base” OR “home-base” OR “health education” OR “patient education” OR “patient participation” OR “health communication” OR “health promotion” OR “self concept” OR “self-control” OR (MH “self concept”) OR “self-regulation” OR “patient autonomy*” OR (MH “patient compliance”) OR (MH “health behavior”) OR “health attitude” OR “illness attitude” OR “patient attitude*” OR (MH “choice behavior”) OR (MH “illness behavior“) OR “self management” “disease management”AND “chronic pain” OR “musculoskeletal pain” OR “myalgia” OR “neck pain” OR “musculoskeletal pains” OR “muscle pain” OR “neck pains” OR “cervical pain” OR “cervical pains” OR “myofascial pain syndrome” OR “trigger point”)	1982

**Table 2:** Characteristics of the studies included.

Study (Year)	Study Design/Groups	Sample per Group Group No (% Women)	Sample Age Years ± SD	Duration of Pain Years ± SD	Pain Intensity (VAS) Mean ± SD 0–10
Javdaneh N. et al. (2021) [36]	RCT/3	SCE + PT: n = 24 (54.16) PT: n = 24 (41.66) CG: n = 24 (50)	SCE + PT: 33.45 ± 7.08 PT: 31.18 ± 6.37 CG: 33.70 ± 8.13	SCE + PT: 3.12 ± 0.85 PT: 3.45 ± 0.84 CG: 3.76 ± 1.17	NR
López-de-Uralde-Villanueva I. et al. (2021) [37]	RCT/3	SCE + PT: n = 16 (81.3) SCE + PT + Ex: n = 16 (68.8) PT: n = 15 (80)	SCE + PT: 38.59 ±16.6 SCE + PT + Ex: 40.94 ± 13.77 PT: 43.53 ± 15.92	SCE + PT: 4.71 ± 5.23 SCE + PT + Ex: 6.89 ± 7.58 PT: 7.98 ± 6.46	SCE + PT: 5.31± 1.66 SCE + PT + Ex: 5.68 ± 1.56 PT: 5.39 ± 2.1
Sihawong R. et al. (2020) [38]	RCT/2	SCE: n = 27 (66.7) Ex: n = 13 (85.7)	SCE: 40.3 ± 10.8 Ex: 41.9 ± 10.1	NR	SCE: 5.8 ± 1.6 Ex: 6.0 ± 2.0
López-López L. et al. (2020) [39]	RCT/2	SCE + PT: n = 27 (NR) PT: n = 26 (NR)	SCE + PT: 38.88 ± 14.01 PT: 40.06 ± 8,32	SCE + PT: 1.64 ± 0.65 PT: 1.78 ± 0.49	SCE + PT: 6.97 ± 1.45 PT: 6.4 ± 2.52
Thompson D.P. et al. (2018) [40]	RCT/2	SCE: n = 29 (41) Ex: n = 28 (50)	SCE: 49.2 ± 14.5 Ex: 45.8 ± 16.6	SCE: 4.8 ± 6.8 Ex: 3.9 ± 7.3	SCE: 5.9 ± 2.1 Ex: 5.4 ± 2.1
Gialanella B. et al. (2017) [41]	RCT/2	SCE + PT: n = 47 (89.3) CG: n = 47 (89.3)	SCE + PT: 56.0 ± 14.0 CG: 60.1 ± 11.0	NR	SCE + PT: 6.8 ± 1.3 CG: 6.6 ± 1.5
Beltran-Alacreu H. et al. (2015) [42]	RCT/3	SCE + PT: n = 15 (86.7) PT1: n = 15 (80) PT2: n = 15 (66.7)	SCE + PT: 40.9 ± 16.2 PT1: 43.5 ± 15.9 PT2: 39.8 ± 13.4	SCE + PT: 4.57 ± 4.75 PT1: 7.98 ± 6.45 PT2: 6.95 ± 7.84	NR
Andersen L.L. et al. (2010) [43]	RCT/3	SCE: n = 66 (87.8) Ex1: n = 66 (87.8) Ex2: n = 66 (87.8)	NR	NR	SCE: 3.5 ± 1.7 Ex1: 3.5 ± 1.7 Ex2: 3.9 ± 2.2
Sherman K.J. et al. (2009) [44]	RCT/2	SCE: n = 32 (68.8) CG: n = 32 (68.8)	SCE: 47.4 ± 12.3 CG: 46.4 ± 11.3	SCE: 7.3 ± 6.9 CG: 7.9 ± 9.4	NR
Klaber-Moffet J.A. et al. (2005) [45]	RCT/2	SCE: n = 139 (62) PT: n = 129 (66)	SCE: 48.8 ± 16.56 PT: 47.8 ± 16.62	SCE: 7.25 ± 5.21 PT: 7.33 ± 5.68	NR
Taimela S. et al. (2000) [46]	RCT/3	SCE + Ex: n = 25 (76) PT: n = 25 (68) CG: n = 26 (69.2)	SCE + Ex: 44.8 ± 9.0 (Female) 36.0 ± 8.0 (Male) PT: 44.0 ± 8.43 (Female) 8.8 ± 7.6 (Male) CG: 47.1 ± 16.8 (Female) 43.2 ± 11.0 (Male)	NR	SCE + Ex: 33.3 ± 23.1 (Female) 23.2 ± 19.1 (Male) PT: 48.8 ± 23.1 (Female) 31.5 ± 24.3 (Male) CG: 45.9 ± 24.7 (Female) 25.9 ± 23.5 (Male)

SD: standard deviation; RCT: randomized controlled trial; n: number of participants; SCE: self-care education; PT: physical therapy; CG: control group; NR: not reported; Ex: exercises.

**Table 3:** Characteristics of interventions.

Study (Year)	Interventions	Self-Care Education Programs	Intervention Duration and Frequency Months/Weeks Days/Week	Modality	Outcomes	Main Results
Javdaneh N. et al. (2021) [36]	SCE + PT: education + physical therapy (therapeutic exercise) PT: physical therapy (therapeutic exercise) CG: no treatment	- Information about condition and/or its management. - Provision of/agreement on specific clinical action plans and/or rescue medication. (Face-to-face interviews + slide presentation)	6 w 1 d/w	Group Supervised On-site	Pain: NPAD. Disability: NPAD. Psychological pain-related variables: FABQ, PCS.	SCE + PT improvement in all variables after the intervention. PT improvement in all variables after the intervention. No significant differences in CG after the intervention. Between-group comparison in favor of SCE + PT group.
López-de-Uralde-Villanueva I. et al. (2021) [37]	SCE + PT: education and behavioral therapy + physical therapy (manual therapy) SCE + PT + Ex: therapeutic patient education + manual therapy + exercises PT: physical therapy (manual therapy)	- Information about condition and/or its management. - Practical support with adherence (medication or behavioral). - Training/rehearsal. For everyday activities (face-to-face treatments + slide presentation + booklet)	4 w 2 d/w	Individual Supervised On-site	Pain: VAS. Psychological pain-related variables: PCS.	Both SCE groups (SCE + PT and SCE + PT + Ex) and PT improved after the intervention. Both SCE groups showed greater improvements than CG globally. Except for pain intensity, the SCE + PT group had similar results to the PT group. Yet, the SCE + MT group showed greater improvements than the PT group in pain catastrophizing. At follow-up (4 months), the SCE + PT + Ex group showed better results than the SCE + PT group in pain assessment.
Sihawong R. et al. (2020) [38]	SCE: education group and behavioral therapy Ex: exercise group	- Information about condition and/or its management. - Provision of/agreement on specific clinical action plans and/or rescue medication. - Training/rehearsal. For everyday activities (Checklist + handbook + booklet)	1 w 5 d/w	Individual Mixed Online	Pain: VAS. Disability: NDI, RMDQ.	Both groups improved after the intervention. No difference between groups in pain intensity or disability in any of the variables.
López-López L. et al. (2020) [39]	SCE + PT: education and behavioral therapy + physical therapy PT: physical therapy	- Information about condition and/or its management. - Regular visits with healthcare professionals. - Training about symptom self-management strategies. - Training about practical self-management activities. - Training about psychological strategies. (Face-to-face education program + face-to-face treatments)	2 d/w	Individual Supervised On-site	Pain: BPI. Disability: NDI. Psychological pain-related variables: FABQ.	Catastrophization, as well as pain and health-related quality of life, improved significantly after the intervention in the SCE + PT group. Between-group comparisons showed significant improvements in favor of the SCE + PT group.
Thompson D.P. et al. (2018) [40]	SCE: education and behavioral therapy Ex: exercise group	- Information about condition and/or its management. - Training/rehearsal. For practical self-management activities (Written information + face-to-face exercise group)	NR	Group Supervised On-site	Pain: NPRS. Pain and disability: NPQ. Psychological pain-related variables: PCS, TSK, CPSS-pf, PVAQ.	Improvement after the intervention in both groups. No significant difference in between-group analysis.
Gialanella B. et al. (2017) [41]	SCE + PT: education and behavioral therapy CG: no treatment	- Information about condition and/or its management. - Regular clinical review. - Monitoring of condition with feedback. - Practical support with adherence (medication or behavioral). - Provision of easy access to advice or support when needed. - Lifestyle advice and support.	6 m 1 d/2 w	Individual Supervised Online (phone calls)	Pain: VAS. Disability: NDI.	Improvement after the intervention in both groups, but significantly more marked in the SCE + PT group in pain intensity and disability.
Beltran-Alacreu H. et al. (2015) [42]	SCE + PT: education and behavioral therapy + physical therapy (therapeutic exercises) PT1: physical therapy (therapeutic exercises) PT2: physical therapy (manual therapy)	- Information about condition and/or its management. - Practical support with adherence (medication or behavioral). - Training/rehearsal for everyday activities. (Face-to-face treatments + slide presentation + booklet)	4 w 2 d/w	Individual Supervised On-site	Disability: NDI. Psychosocial pain-related variables: TSK, FABQ.	For neck disability, the CG obtained statistically significant changes in the short and medium term. The FABQ showed statistically significant differences in SCE + PT and PT1 groups but not in PT2.
Andersen L.L. et al. (2010) [43]	SCE: education and behavioral therapy Ex1: 2 m exercise group Ex2: 12 m exercise group	- Information about condition and/or its management. - Practical support with adherence (medication or behavioral). - Training about symptoms self-management strategies. - Training about practical self-management activities. (Written information)	10 w SCE:1 d/w Ex1, 2: 3–5 d/w	IndividualUnsupervised Online (emailed information on various aspects of general health)	Pain: Nordic Questionnaire.	Pain intensity decreased in Ex1 and Ex2 groups, compared to the SCE group, but change was not significant.
Sherman K.J. et al. (2009) [44]	SCE: education and behavioral therapy CG: massage	- Information about condition and/or its management. - Practical support with adherence (medication or behavioral). - Training about symptom self-management strategies. - Training about practical self-management activities. (Written information + handbook)	10 w	Individual Mixed Online	Pain and disability: NPQ. Copenhagen Neck Functional Disability Scale.	At 10 weeks, more participants randomized to massage experienced clinically significant improvement on the NDI and on the symptom bothersomeness scale.
Klaber Moffet J.A. et al. (2005) [45]	SCE: education and behavioral therapy PT: physical therapy	- Information about condition and/or its management. - Practical support with adherence (medication or behavioral) rescue medication. (Face-to-face interviews + booklet)	1 d (can be extended to a maximum of 3)	Individual Mixed On-site	Pain and disability: NPQ. Psychosocial pain-related variables: TSK.	No significant difference between groups.
Taimela S. et al. (2000) [46]	SCE + Ex: education and behavioral therapy + exercises PT: physical therapy (therapeutic exercises) CG: no treatment	- Information about condition and/or its management. - Practical support with adherence (medication or behavioral). - Training/rehearsal for everyday activities. (Written information + face-to-face exercise group + diary of progress)	12 w 2 d/w	Group Mixed On-site	Pain: VAS. Psychological pain-related variables: FABQ.	Significant differences between groups in favor of the experimental group.

W: weeks; d: days; SCE: self-care education; PT: physical therapy; CG: control group; Ex: exercises; NPAD: Neck Pain and Disability Scale; FABQ: Fear-Avoidance Beliefs Questionnaire; PCS: Pain Catastrophizing Scale; VAS: Visual Analogue Scale; NDI: Neck Disability Index; RMDQ: Roland–Morris Disability Questionnaire; BPI: Brief Pain Inventory; NPRS: Numeric Pain Rating Scale; NPQ: Neuropathic Pain Questionnaire; TSK: Tampa Scale for Kinesiophobia; CPSS-pf: Chronic Pain Self-Efficacy Questionnaire—physical function subscale; PVAQ: Pain Vigilance and Awareness Questionnaire; NR: not reported.

**Table 4:** Self-care components in each article included.

Study (Year)	Addressing Physical and Psychological Symptoms	Engaging in Self-Care Strategies	Monitoring and Recording Symptoms	Discussing Symptoms with Providers of Medical Care
Javdaneh N. et al. (2021) [36]	Yes	Yes	No	No
López-de-Uralde-Villanueva I. et al. (2021) [37]	Yes	Yes	No	No
Sihawong R. et al. (2020) [38]	Yes	Yes	Yes	No
López-López L. et al. (2020) [39]	Yes	Yes	No	Yes
Thompson D.P. et al. (2018) [40]	Yes	Yes	No	No
Gialanella B. et al. (2017) [41]	Yes	Yes	Yes	Yes
Beltran-Alacreu H. et al. (2015) [42]	Yes	Yes	No	No
Andersen L.L. et al. (2010) [43]	Yes	Yes	No	No
Sherman K.J. et al. (2009) [44]	Yes	Yes	No	No
Klaber-Moffet J.A. et al. (2005) [45]	Yes	Yes	No	No
Taimela S. et al. (2000) [46]	Yes	Yes	Yes	No

## Data Availability

Data supporting this systematic review are available in the original publications, reports and preprints that were cited in the reference section. In addition, the analyzed data that were used during this systematic review are available from the author upon reasonable request.
